# Childhood overweight in Berlin: intra-urban differences and underlying influencing factors

**DOI:** 10.1186/s12942-016-0041-0

**Published:** 2016-03-22

**Authors:** Tobia Lakes, Katrin Burkart

**Affiliations:** Geography Department, Humboldt-Universität zu Berlin, Unter den Linden 6, 10099 Berlin, Germany; Department of Environmental Health Science, Mailman School of Public Health, Columbia University in the City of New York, New York, NY USA

**Keywords:** Obesity, Overweight, Childhood, Neighbourhood, Urban health, Spatial analyses

## Abstract

**Background:**

In recent years, childhood overweight and obesity have become an increasing and challenging phenomenon in Western cities. A lot of studies have focused on the analysis of factors such as individual dispositions and nutrition balances, among others. However, little is known about the intra-urban spatial patterns of childhood overweight and its associations with influencing factors that stretch from an individual to a neighbourhood level. The aim of this paper is to analyse the spatial patterns of childhood obesity in Berlin, and also to explore and test for associations with a complex set of risk factors at the individual, household and neighbourhood levels.

**Methods:**

We use data from a survey of 5–6 year-olds that includes health status, height, and weight, as well as several socioeconomic and other risk variables. In addition, we use a set of neighbourhood variables, such as distance, and density measures of parks or fast food restaurants. Our outcome variable is the percentage of children of 5–6 years who were reported overweight or obese in 2012. The aggregated data is available for 60 areas in Berlin. We first analyse the outcome and risk factor data descriptively, and subsequently apply a set of regression analyses to test for associations between reported overweight and obesity, and also individual, household and neighbourhood characteristics.

**Results:**

Our analysis returned a distinct spatial distribution of childhood overweight in Berlin with highest shares in the city centre. Moreover, we were able to identify significant effects regarding the social index, and the percentage of non-German children being obese or overweight; additionally, we identified fast food restaurant density as a possible influencing factor. For the other variables, including the neighbourhood variables, we could not identify a significant association on this aggregated level of analysis.

**Conclusions:**

Our findings confirm the results of earlier studies, in which the social status and percentage of non-German children is very important in terms of the association with childhood overweight and obesity. Unlike many studies conducted in North America, this study did not reveal an influence of neighbourhood variables. We argue that European urban structures differ from North American structures and highlight the need for a more detailed analysis of the association between the neighbourhood environment and the physical activity of children in urban setting.

**Electronic supplementary material:**

The online version of this article (doi:10.1186/s12942-016-0041-0) contains supplementary material, which is available to authorized users.

## Background

Childhood overweight and obesity have become a major global public health concern, since they are closely associated with cardiovascular disease, diabetes, asthma, cancer, and psychological distress which create enormous health care costs as a consequence [1–4]. From earlier studies, it is known that a complex set of factors from the individual, to the household and neighbourhood levels is associated with childhood obesity. Han et al. [5] provide a systematic overview of the determinants of obesity among children in the 2010 Lancet seminar on childhood obesity. Existing knowledge on influencing factors focuses particularly on individual level risk factors such as an individual’s disposition, diet, energy supply, genetic history, psychology, socio-cultural relationship, education, nutrition balances, etc. [6].

While individual and household factors have been identified as very important for overweight and obesity in general, there are very few studies on the relevance of neighbourhood factors [1, 2, 4]. However, modifying energy imbalances that lead to overweight and obesity in urban populations will require targeting the obesogenic environment in particular, in addition to reducing caloric intake [2, 7, 8]. Although some evidence states that more walkable neighbourhoods are associated with more physical activity and/or less obesity [2, 4, 9–14], Feng et al. [15] could not state a clear, consistent association between the built environment and obesity in their systematic review. Moreover, most of the literature on the impact of neighbourhoods on obesity focuses on adult populations. Specific evidence on the association between neighbourhoods and childhood obesity is scarce [4, 10, 16]. Regarding physical activity in the everyday lives of children, we know that they are particularly affected by domestic living situations, in addition to the built and physical environment in the immediate neighbourhood [17]. This statement holds true for preschool children in particular, because of their limited range of activity, and the dependency they have on their parents and family for mobility.

Generally, the concept of neighbourhood walkability is commonly used to describe the influence of the built environment on physical activity. Walkability is mainly quantified by indicators such as the IPEN Walkability index [18] or the walk score (https://www.walkscore.com/), which considers indicators such as residential density, street connectivity, the availability of walkable destinations, and land-use mix (residential, commercial, retail, recreation) [1, 4, 11, 12]. Additional qualitative aspects of a physically active neighbourhood (e.g. crime/traffic safety) have only recently been considered [4, 12, 16, 19–21].

Up to now, most of the studies concerning physically active neighbourhoods and obesity originate from urban settings in the United States. European cities exhibit different city structures, and in this case the association between walkability and obesity has only rarely been investigated [1, 22, 23]. Spatial planning that supports physical activity, however, becomes a major challenge in childhood obesity measures [24]; for example, [25] identifies the following environmental characteristics as decisive for the physical activity of children: access to space for physical activity (parks and playgrounds, physical activity centers, swimming pools etc.); urban neighbourhood settings (living in the city center, high heterogeneity of urban land use, high street connectivity); road traffic and safety (low road traffic, availability of traffic lights, pedestrian paths, bike lanes, public transport); social living environment (perceived safety, low crime rates).

To enable health-oriented spatial planning and the allocation of resources for public health measures in urban areas, a detailed knowledge of the spatial patterns and influencing factors is required; not only at the individual and household levels, but also the neighbourhood level. Therefore, the aim of this paper is to analyse the intra-urban patterns of childhood overweight and obesity in Berlin, and to explore and test for associations with possible influencing factors from the individual to the neighbourhood level.

## Methods

### Study area and data

In this study, we focus on Berlin, the capital of Germany, which has a total population of about 3.5 million inhabitants in an area of circa 892 km^2^. Berlin is characterized by its highly heterogeneous structure in terms of residential living environments, built urban neighbourhoods, and socio-economic characteristics. The urban structure varies widely across the 12 districts, and includes densely populated areas in the city center, as well as single family homes in the suburban areas. The same holds true for the distribution of parks and open spaces. Regarding the distribution of socio-economic status, we generally identify higher levels in the suburban areas and lower levels in the city center, with a few exceptions.

For our analysis, we used the datasets described in Table 1. We chose the percentage of overweight and obese preschool children (5–6 years) from 2012 as an outcome variable. This variable was derived from an annual preschool examination mandatory for all children with a residency in Berlin. In total, a number of 28,159 children were surveyed in 2012. Overweight and obesity were defined according to the reference values of Kromeyer-Hauschild following the suggestions of the working group Adipositas (Arbeitsgemeinschaft Adipositas) [26]. According to these age- and gender-specific thresholds, overweight children are classified from the 90–97th percentile and obese children above the 97th percentile. The available dataset is already preprocessed and aggregated due to privacy issues on the level of 60 areas [26]. These 60 areas were not only comparable in terms of area and population numbers, but were also created in an inter-organisational discussion process to reflect homogeneity in living environments; for example, by focusing on unique built structures and social milieus, we can expect similar neighbourhood and socioeconomic conditions within the areas for our analysis. As predictor variables we chose the individual and household level determinants that were reported in the child survey. We build upon existing knowledge on the influencing factors of overweight and obesity [5] and use the following explanatory factors: language skills, health status, availability of a TV, kindergarten attendance, migration background, and the social index that combines parents’ education, and employment status (see Table 1). However, additional information was not available on individual-level genetic disposition and nutrition information, among other criteria.

**Table 1:** Outcome and predictor variables: descriptive statistics (mean, median, standard deviation and relative standard deviation)

Variable	Mean	Median	Standard deviation	Relative standard deviation
*Child survey*				
Percentage of overweight and obese pre-school children (5–6 years) (dependent variable)	9.31	8.60	4.19	45.02
Social index (based on parents education and employment status)	13.97	14.00	1.95	13.97
Percentage of non-German children	35.78	33.40	22.57	63.09
Percentage of non German children with insufficient German language skills	8.29	6.50	8.34	100.51
Percentage of children with measles vaccination	91.15	92.40	4.18	4.59
Percentage of children with bad dental hygiene	12.39	11.20	7.24	58.41
Percentage of children participating in regular medical check-ups	87.67	88.00	4.68	5.34
Percentage of children living in household with at least one smoking parent	36.03	37.20	10.72	29.75
Percentage of children having their own TV in bedroom	11.45	10.10	7.06	61.63
Percentage of children with poor eye-hand coordination	15.63	15.40	6.80	43.50
Percentage of German children with poor language skills	11.85	9.30	8.48	71.61
Percentage of non-German children with poor language skills	20.80	20.70	13.14	63.18
Percentage of children attending Kindergarten	89.82	90.20	5.21	5.80
*Environmental variables and urban characteristics*				
Percentage of area with vegetation	0.22	0.17	0.16	71.25
Area of parks per km^2^	7.1 × 10^−2^	5.5 × 10^−2^	5.2 × 10^−2^	73.23
Number of parks per km^2^	2.2 × 10^−3^	2.2 × 10^−3^	1.2 × 10^−2^	55.39
Area of parks per 1000 inhabitants	15.7 × 10^3^	11.6 × 10^3^	13.9 × 10^−3^	89.21
Number of parks per 1000 inhabitants	0.00	0.00	0.00	67.86
Euclidean distance to parks	429.16	330.27	326.13	75.99
Area of playgrounds per km^2^	6.5 × 10^−3^	4.6 × 10^−3^	5.9 × 10^−3^	90.82
Number of playgrounds per km^2^	3.7 × 10^−3^	2.2 × 10^−3^	3.5 × 10^−3^	95.53
Area of playgrounds per 1000 inhabitants	9.1 × 10^2^	8.7 × 10^2^	3.7 × 10^2^	40.43
Number of playgrounds per 1000 inhabitants	5.1 × 10^−1^	4.9 × 10^−1^	1.8 × 10^−1^	34.60
Euclidean distance to playgrounds	488.96	359.74	403.17	82.45
Number of fast food restaurants per km^2^	3.8 × 10^−6^	1.5 × 10^−6^	5.0 × 10^−6^	132.29
Number of fast food restaurants per 1000 inhabitants	4.3 × 10^−1^	3.6 × 10^−1^	2.8 × 10^−1^	65.05
Availability of public transport	1134.74	868.42	914.95	80.63
*Walkability*				
Connectivity	−0.04	−0.09	1.00	−2377.29
Entropy	−0.10	0.03	1.00	−1012.31
Population density	−0.03	−0.34	1.00	−3948.53

In addition, we include determinants that describe the neighbourhood characteristics in environmental and urban terms, as well as the walkability of the area. We choose vegetation, parks, playgrounds, and fast food restaurants as possible sites of interest that are relevant to childhood overweight and obesity. We then calculate different measures of area and distance: the percentage of area, the area per km^2^, the number of features, and the Euclidian distance for those. To additionally include a measure of public mobility, we calculated the availability of public transport access points. Moreover, we used data on the walkability of the neighbourhood according to an adapted version of the Walkability Index [18]. The following components of the index were assessed: street connectivity, residential density, and land use mix. Connectivity was represented by the intersection density derived from the street network. To capture residential density, population density was used. Households numbers were estimated by using the average Berlin household size of 1,7 persons per household. An entropy index indicating the evenness of distribution of different land uses was used to estimate land use mix. The original IPEN index further includes a floor ratio to estimate the retail area [18] which may be possible destinations to walk or bike to. Yet, that component of the index was left out because in an European context it may overestimate the actual retail area, as in contrast to land use patterns in the US, European land use is shaped by mixed uses within one building, which are either classified as retail or non-retail, thereby leading to biased data. The datasets were derived from the Senate Department of Urban Development [27] and from OpenStreetMap (2014).

### Analysis

We first plotted the target variable percentage of obese preschool children (age 5–6) on the level of the 60 areas.

### Descriptive statistics and correlation analysis

We calculated the mean and median, as well as the standard and relative deviation of our outcome and predictor variables to understand the general distribution and variation. In order to better understand the internal correlation structure of our explanatory variables, we conducted a correlation analysis. Variables considered are such as listed in Table 1.

### Regression modelling

We used parametric (linear) as well as non-parametric regression models in order to understand the influence of spatial characteristics on the spatial distribution of overweight and obesity in preschool children. We chose a stepwise regression approach in order to select predictor variables. Our approach consisted of forward selection as well as bidirectional selection (we did not work with backward elimination, as the set of potential predictor variables was too extensive to fit a model including all predictors). We primarily fitted univariate regression models and determined the model fit and predictive power of each particular variable. The variable showing the highest predictive power was included into the model in the first step. Subsequently, variables showing a significant effect, in addition to those that best improved the model fit (based on adjusted R^2^) were included. Variables that did not have a significant influence after including another predictor variable were eliminated from the model as part of the bidirectional selection process. To deal with spatial autocorrelation, we included x- and y- coordinates of the unit centroids into our models. As in the final model, these coordinates did not show to be significant anymore; as a result, they were excluded from the final analysis. All analysis was carried out with R (Version 0.98.1102) using generalized additive models (package “mgcv”).

Initially, we fitted splines (non-parametric), allowing for non-linear relationships between the outcome and the predictor variable. As relationships could be sufficiently described in linear terms, we subsequently fitted linear (parametric) models and quantified the effect of the selected predictor variables. Effect estimates, i.e., % changes per unit change in the predictor were calculated as follows:

$$\% change = (e^{\beta } - 1) \times 100.$$

## Results

### Intra-urban patterns of childhood overweight and obesity in Berlin

**Fig. 1:**
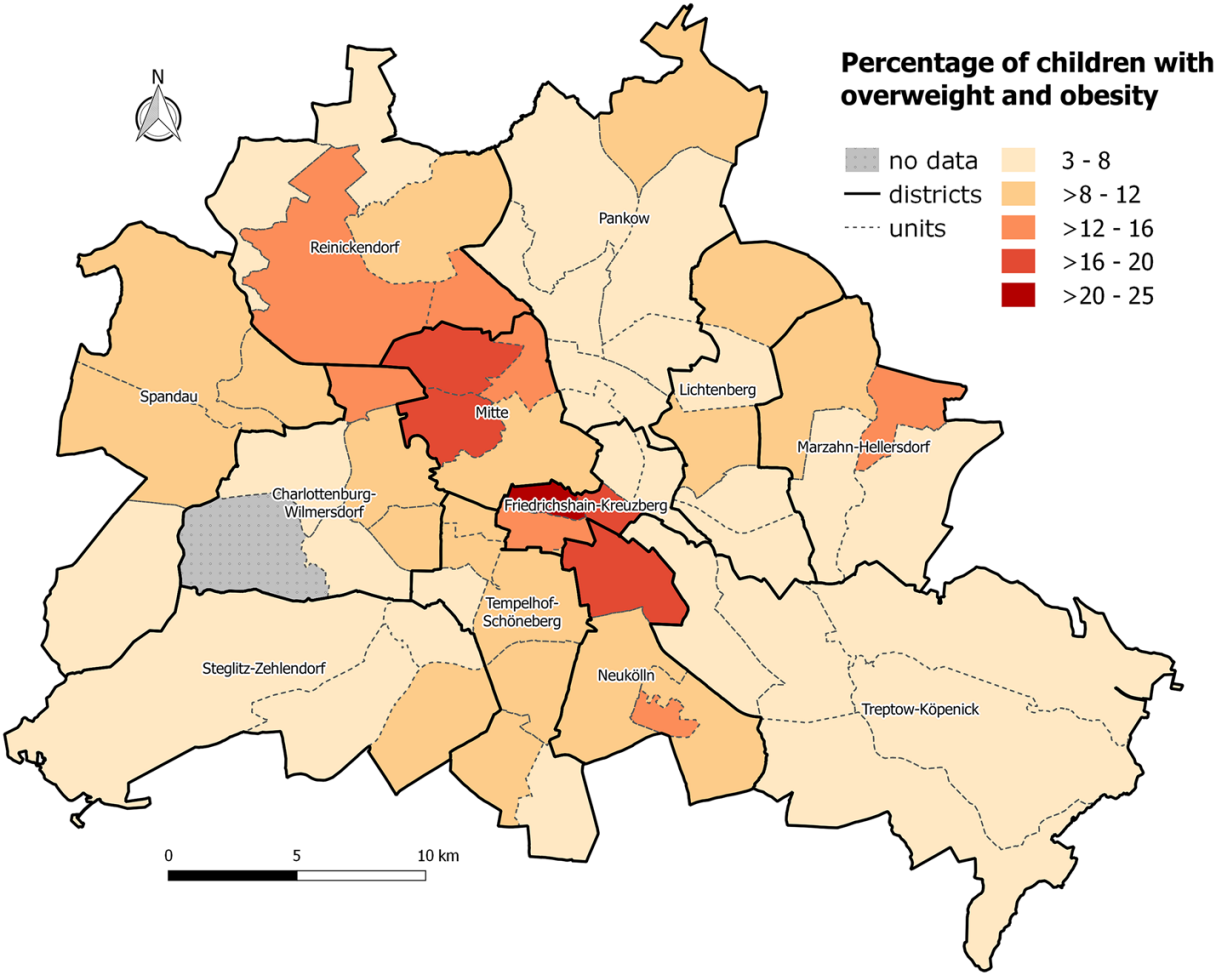
Intra-urban patterns of overweight and obesity in pre-school children in Berlin

The spatial distribution of childhood overweight and obesity is shown in Fig. 1. A distinct intra-urban pattern could be identified, with the highest share of overweight and obesity in the inner city in areas in the neighbourhoods of Mitte, Friedrichshain-Kreuzberg and Neukölln, showing more than 16 % of overweight and obese children. On the contrary, the suburban areas are characterized by low numbers of reported overweight and obesity, with less than 8 %. What becomes evident in the depiction of the rates is that it is a spatial phenomenon that varies very much across the city with values between 3.2 % (Southern Prenzlauer Berg) and 24.9 % (Kreuzberg-North).

### Distribution of population and neighbourhood characteristics

Similar to overweight and obesity, the descriptive analysis of possible influencing factors revealed a heterogeneous pattern and distribution in the city of Berlin. Table 1 exhibits standard descriptions of the predictor variables (mean, median, standard deviation and relative standard deviation). For variables collected within the child survey, the mean and median variables were close, while for environmental variables there were rather pronounced differences. Relative standard deviations ranged between 5 % for kindergarten attendance, yet displayed markedly higher values for the percentage of non-German children per unit, in addition to other spatial variables such as vegetation cover, access to public transport, area, the number of parks and playgrounds, or fast food restaurant density. Particularly high standard deviations were found for walkability indices (cf. Table 1).

The correlation analysis revealed a strong association between social index, the percentage of non-German children, and several other variables collected within the child survey. In areas with a low social index, the share of non-German children was significantly higher. Generally, in areas with a high social index, the share of children having bad teeth and language deficits, or showing poor eye-hand coordination was smaller. Moreover, parents were less likely to smoke and fewer children had their own TV. Furthermore, the number of children attending kindergarten and participating in regular medical check-ups increased. Areas with a high percentage of non-German children showed a higher incidence of language deficits, and children were less likely to attend kindergarten or participate in regular medical check-ups. The share of children having a weak dental condition was higher and the possibility of having a smoking parent and a TV moderately increased.

While there was no association between social index and vegetation, or access to public transport, areas with a high percentage of non-German children show a moderately negative correlation with both variables. With regard to walkability, there was no clear tendency: outcomes depended on the walkability indicator. All walkability indices are positively correlated with the percentage of non-German children. Regarding social status, we find a contradictory result: connectivity is positively correlated, while entropy and population are negatively correlated with social status. Regarding the number and area of parks and playgrounds, we did not find an association with social index, but did find that areas with a high share of non-German children showed an increased area and number of parks per unit, while also showing a smaller population per unit. A similar pattern was found for playgrounds. The distance to parks and playgrounds decreased with the increase in the share of non-German children, while there was no relationship with social index.

### Associations between childhood overweight and obesity and influencing factors

**Fig. 2:**
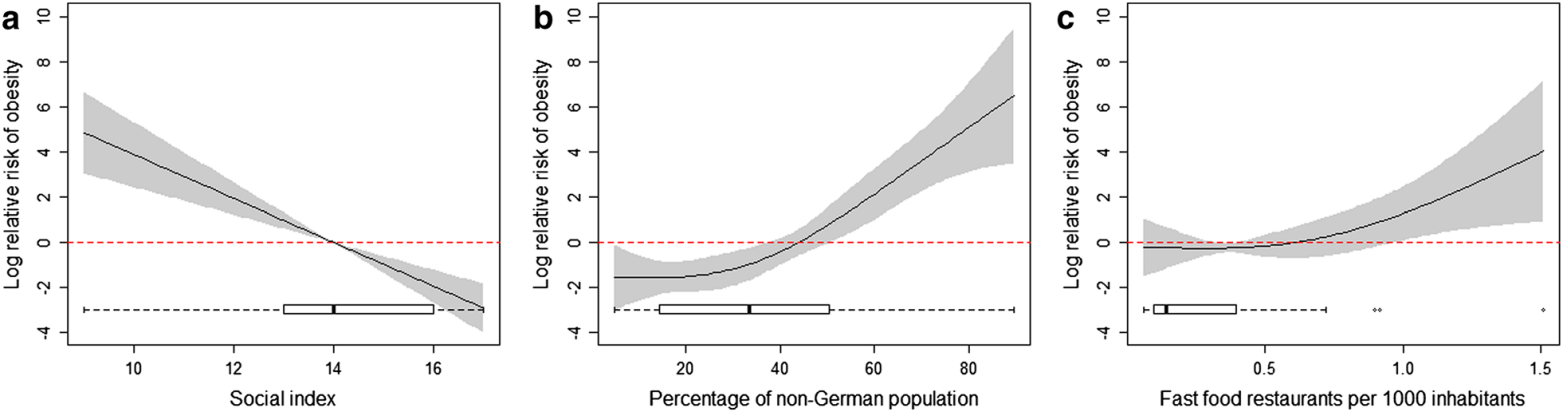
Relationship between overweight and obesity and social index (**a**) percentage of non-German children (**b**) and fast food restaurants density (**c**). *Grey areas* depict 95 % confidence intervals. *Boxplots* of the particular predictor variable are shown at the *bottom* of each *plot*

**Fig. 3:**
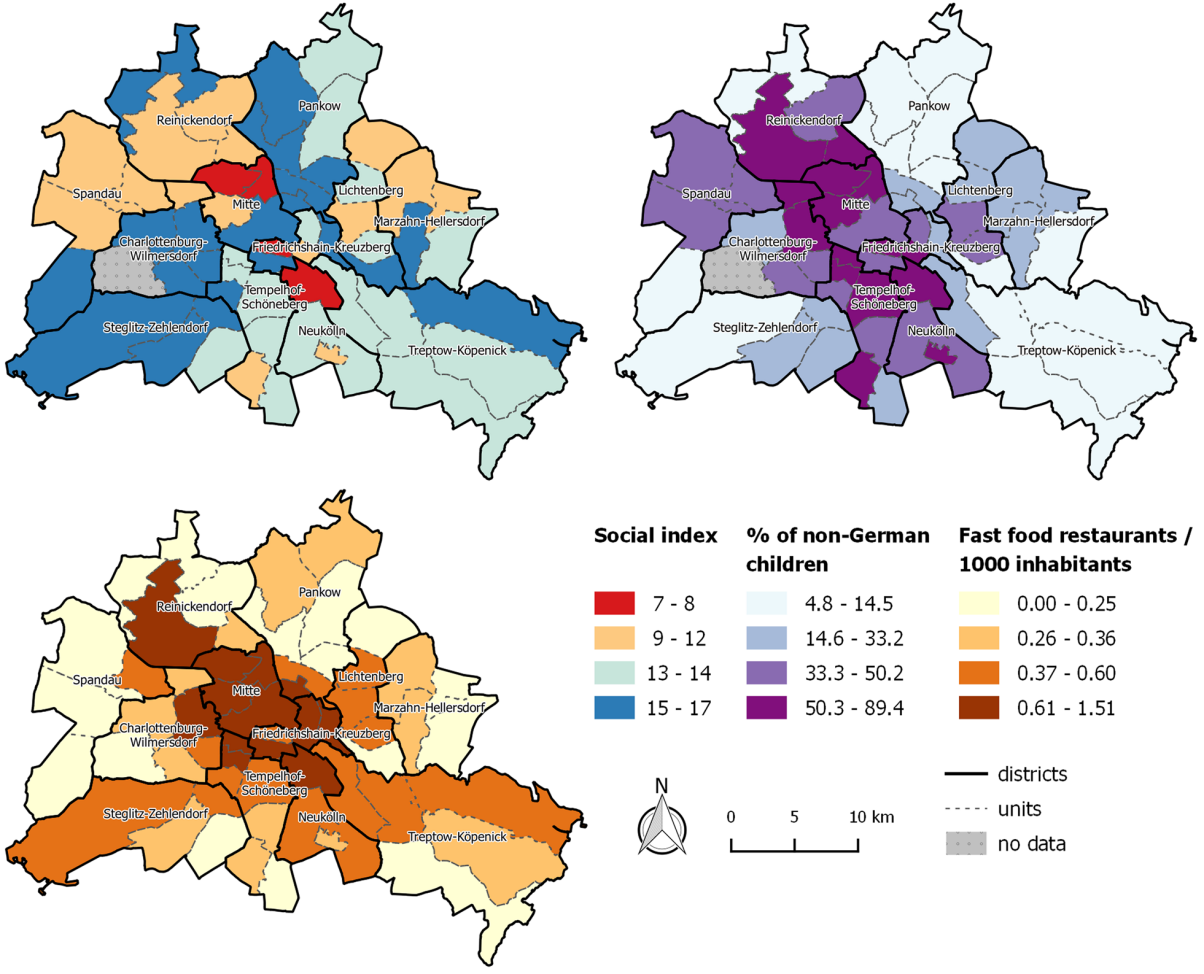
Intra-urban patterns of social index, percentage of non-German children and fast food restaurants density

The final three variables that were selected through the selection process as described in “Descriptive statistics and correlation analysis” section were: social index, percentage of non-German children and fast food restaurant density. Figure 2 depicts the influence of these three predictors on overweight and obesity in preschool children in Berlin from the multivariate non-parametric regression model. In addition, we present the underlying distribution of the three variables in Fig. 3, while the other, non-significant, ones are given in the Additional file 1: S1.

We found a negative association between social index and child overweight and obesity indicating that the risk of being overweight/obese increased with decreasing social status in a unit. Moreover, we found a positive association between percentage of non-German children per unit and overweight/obesity as well as between fast food restaurant density and overweight/obesity. The overall explained deviance (R^2^-adjusted) amounted to 81.2 % for the non-parametric modelling approach with this final model.

When fitting a linear model with social index, the percentage of non-German children and fast food restaurant density the explained deviance (R^2^-adjusted) was 78.8 % (Table 2).

**Table 2:** Outputs of multivariate regression models on influencing factors of overweight/obesity in Berlin

	β_0_ (Intercept)	β_1_ (Social index)	β_2_ (Percentage of non-Germans)	β_3_ (Fast food restaurants per 1000 inhabitants	R^2^-adjusted (%)
Model 1	21.7	−1.2	0.07	2.5	78.8
*p* values	2.1 × 10^−10^	3.3 × 10^−8^	8.5 × 10^−5^	0.03	
Model 2	22.0	−1.1	0.07	1.5^a^	79.2
*p* values	1.3 × 10^−9^	2.0 × 10^−7^	4.0 × 10^−4^	0.38	
Model 3	19.7	−1.0	0.1		77.2
*p* values	1.5 × 10^−9^	2.6 × 10^−7^	1.4 × 10^−8^		

^a^Outliers (i.e., areas with fast food restaurant density of above 0.8 restaurants per 1.000 inhabitants) were excluded from analysis

Fast food restaurant density had a significant influence on overweight and obesity, however, when looking at the variable distribution (as indicated by the boxplot in Fig. 2c) we see that values at the upper end are small, and can thus be considered as outliers. When excluding areas with a fast food restaurant density of above 0.8 restaurants per 1000 inhabitants, we still observed an increase in overweight and obesity with increasing fast food restaurant density, although the effect is not significant. Fitting models excluding fast food restaurant density and only including social index and the percentage of foreigners resulted in an explained deviance of 77.2 % for the linear models.

Table 3 displays the percentage change in overweight and obesity per unit change in a particular predictor variable: an increase of 1 point in social index (total range of social index: 4–18) results in a −68.5 % (95 % CI −77.8 to −55.2 %) decrease in overweight and obesity in a unit; while an increase in 1 % in foreigners is associated with a 7.7 % (95 % CI 4.1–11.5 %) increase in child overweight and obesity. An increase in the fast food restaurant density of 0.1 restaurants per 1000 inhabitants would lead to an increase of 30.0 % (2.7–59.6 %) in child overweight and obesity (% change exemplified for Model 1).

**Table 3:** Percentage change in overweight/obesity per unit increase in predictor variables. 95 % confidence intervals are displayed in brackets

	% Change in obesity per		
	1 Point increase in SI	1 % Increase in non-German children	0.1 Fast food restaurant increase per 1000 inhabitants
Model 1	−68.5 % (−77.8 to −55.2 %)	7.7 % (4.1–11.5 %)	30.0 % (2.7 to 59.6 %)
Model 2	−68.1 % (−78.0 to −53.8 %)	7.4 % (3.5–11.4 %)	36.1 % (−8.5 to 139.3 %)^a^
Model 3	−62.9 % (73.4 to 48.3 %)	10.2 % (7.1–13.4 %)	

^a^Outliers (i.e., areas with fast food restaurant density of above 0.8 restaurants per 1.000 inhabitants) were excluded from analysis

## Discussion

This study demonstrated that there are distinct spatial differences in the distribution of child obesity within the city of Berlin. A large share of this intra-urban distribution may, to a great extent, be explained by spatial differences in social status and the percentage of non-German children. Our findings are in-line with an earlier report that presented obesity prevalence for different social status [26] and highlighted the strong association between social status and the prevalence of overweight and obesity. Other studies have demonstrated an increased risk of obesity in children from low social status groups in Germany and Europe as a whole [22, 28–32]. Biological as well as behavioural aspects have been cited as explanations: Parental and maternal overweight in particular are identified as strong risk factors for childhood obesity [33–36]. Moreover, there is a well-established association between low socio-economic status and poor dietary habits. Children in low income households tend to eat less fruits and vegetables, but more sugar, fats, processed meat, and soft drinks [37, 38]. Some researchers also postulate a relationship between low socio-economic status and a lack of physical activity in young people [22, 39–43].

The social status may be closely linked to the number of non-German children that we identified as another important and significant explanatory factor. Our findings are supported with an earlier analysis [26] where 4.4 % of German children were overweight and 2.4 % obese, whilst children with Turkish migration background exhibited an overweight rate of 10.7 %, those of Arab background 9.1 % and those from Eastern European countries 7.3 % (obesity rates were 10.1, 7.6 and 5.3 %). Among children from other western industrial countries 2.6 % were overweight, while an equally high percentage were obese. Similarly, other authors have reported a higher prevalence in children of non-German nationality [44, 45]. Moß et al. [44] noted that overweight and obesity occurred twice as often in non-German as compared to German children, with the highest prevalence present in Turkish children. A study from Aachen [45] found that the prevalence of most known risk factors for overweightness, such as low physical activity, a high consumption of soft drinks, and frequent visits to fast-food restaurants was higher in the children with other nationalities than in the German children. While we were unable to address these underlying behavioural factors, or obtain detailed data on migration background or actual dietary intake, we found a significant association between children of non-German backgrounds and the risk of overweight and obesity.

Spatial and environmental characteristics, such as urban vegetation, parks, playgrounds, access to public transport, or walkability did not show a significant effect on overweight and obesity on the aggregated level of analysis in pre-school children in Berlin. The regression analysis revealed that fast food restaurants had a small impact with increasing overweight and obesity in areas with a higher density of restaurants offering fast food, though the validity of this finding is disputable. Other studies implicate a stronger effect between the availability of fast food restaurants and overweight/obesity; although the picture is not clear, it seems to be differentiated according to childhood age groups [46, 47]. We must also consider that similar to the study in the UK [47], the number of fast-food restaurants is higher in Berlin, particularly in areas with lower socio-economic status. While availability to public transport did not show any significant connection to overweight and obesity in our study, others have shown that independent from the degree of neighbourhood walkability, those who walked to public transport showed more physical activity than nonusers [40]. Few studies have found a direct effect, and some have even identified to the contrary, in that living in a high-walkable neighbourhood was associated with higher levels of sedentary time [48]. Our findings did not reveal a significant effect of walkability measures in the neighbourhood on overweight and obesity. This may be partly explained by the nature of our study: First, we had to rely on the aggregated data on the level of 60 areas of Berlin; and second, we were not able to capture the individual actual mobility and behaviour, but only the availability of public transport or playgrounds for possible use etc.

The multitude of studies so far have been conducted in North America with only a few studies in European settings such as Germany, the Netherlands, Belgium or the UK [9, 10, 16, 22, 47, 48]. While studies conducted in North America highlight the relevance of the environmental influences such as walkability/bikeability, mixed land use, accessible destinations, transit or access to high-caloric foods our study did not confirm such an association in Berlin [4, 9, 10, 14]. Spatial urban (and suburban) structures in Europe and in Germany might differ strongly from the environments investigated in North America. The, so far, limited number of European studies draws an inconclusive picture: A study analyzing the relationship between individually assessed walkability and active transportation in Stuttgart, Germany showed that the more walkable an area was, the more active residents were [23]. However, a Belgian study drew the opposite conclusion: living in a high-walkable neighbourhood was associated with higher levels of sedentary time [48]. Two studies assessing child as well as adult obesity in Kiel, Germany found a rather small influence of environmental characteristics [16, 22]. Gose et al. [22] highlighted the relevance of familial/social factors over neighbourhood environment for childhood obesity and Lange et al. [16] concluded that recommendations for structural policy measures as part of prevention of overweight in adolescents must be made cautiously.

Most of the studies assessing the influence of environmental factors followed a cross sectional design and analyzed data collected from individual study participants. In contrast, our study relied on aggregated data on the ecological level. Aggregation always bears a loss of information and may partly explain the low association between neighborhood environment and obesity in this study. However, a recent review [15] also showed that the evidence about associations between obesity and influencing factor is very controversial. In a comprehensive review, the authors showed that for the studies undertaken no clear picture about the effect of neighborhood environment can be drawn. Hill et al. [8] stated in their publication “Biology clearly contributes to individual differences in weight and height, but the rapid weight gain that has occurred over the past three decades is a result of the changing environment”. The neighbourhood environment and particularly the overconsumption of energy due to availability of energy-dense foods are referred to as decisive factor as well as the modified physical activity behaviours. Information about energy balances to change the behaviour of individuals is therefore one major task for the future [8] in addition to developing urban environments that foster physical activity of children. Detailed individual behaviroural data using also GPS- and activity- assessments may be of large benefit for future in-depth studies (see also [15]). However, at the same time assessments using aggregated data is generally more accessible and available for larger scale areas. Thus, the research approach we are presenting can easily be adapted to other areas—a challenge which was called for in the recent publication [15]. This allows regional and cross-country comparisons which seem highly crucial for European settings.

## Conclusions

The study mapped the childhood overweight and obesity in Berlin and showed a distinct intra-urban variation with high numbers in the centre and lower numbers of overweight and obesity in the suburban areas. Moreover, we tested for associations with a complex set of risk factors from the individual to the household and neighbourhood levels. Our analysis confirms earlier studies in the significant effect of social index and percentage of non-German children on overweight and obesity. In addition, we highlight the effect of availability of fast food restaurants. For the other variables, including the neighbourhood variables, we cannot identify a significant association. From our Berlin findings we can confirm the earlier studies, but moreover, we need to conclude that there is a high demand for a more detailed analysis of the association between the neighbourhood environment and the actual physical activity of children. Our findings call for differentiated health measures to address the problem of obesity in the complex urban setting in a locally-adapted way [49]. Prevention and intervention programs to address childhood overweight and obesity can benefit from spatial insights for prioritizing the most important areas within a city. Furthermore, by taking the socio-economic and cultural influencing factors of overweight and obesity into account, strategies should be developed in close collaboration with community members that specifically address the identified target groups at risk [50]. We therefore conclude with [51], in that we can only develop adequate and new decision-making guidelines once we have understood the associations between the neighbourhood characteristics and obesity in urban areas.

## Additional file

10.1186/s12942-016-0041-0 Intra-urban patterns of studied influencing factors on overweight and obesity.
