# Supplementary figures and images for: Childhood overweight in Berlin: intra-urban differences and underlying influencing factors
Source: Int J Health Geogr. 2016 Mar 22;15:12. doi: 10.1186/s12942-016-0041-0 (PMC4802651; doi:10.1186/s12942-016-0041-0)

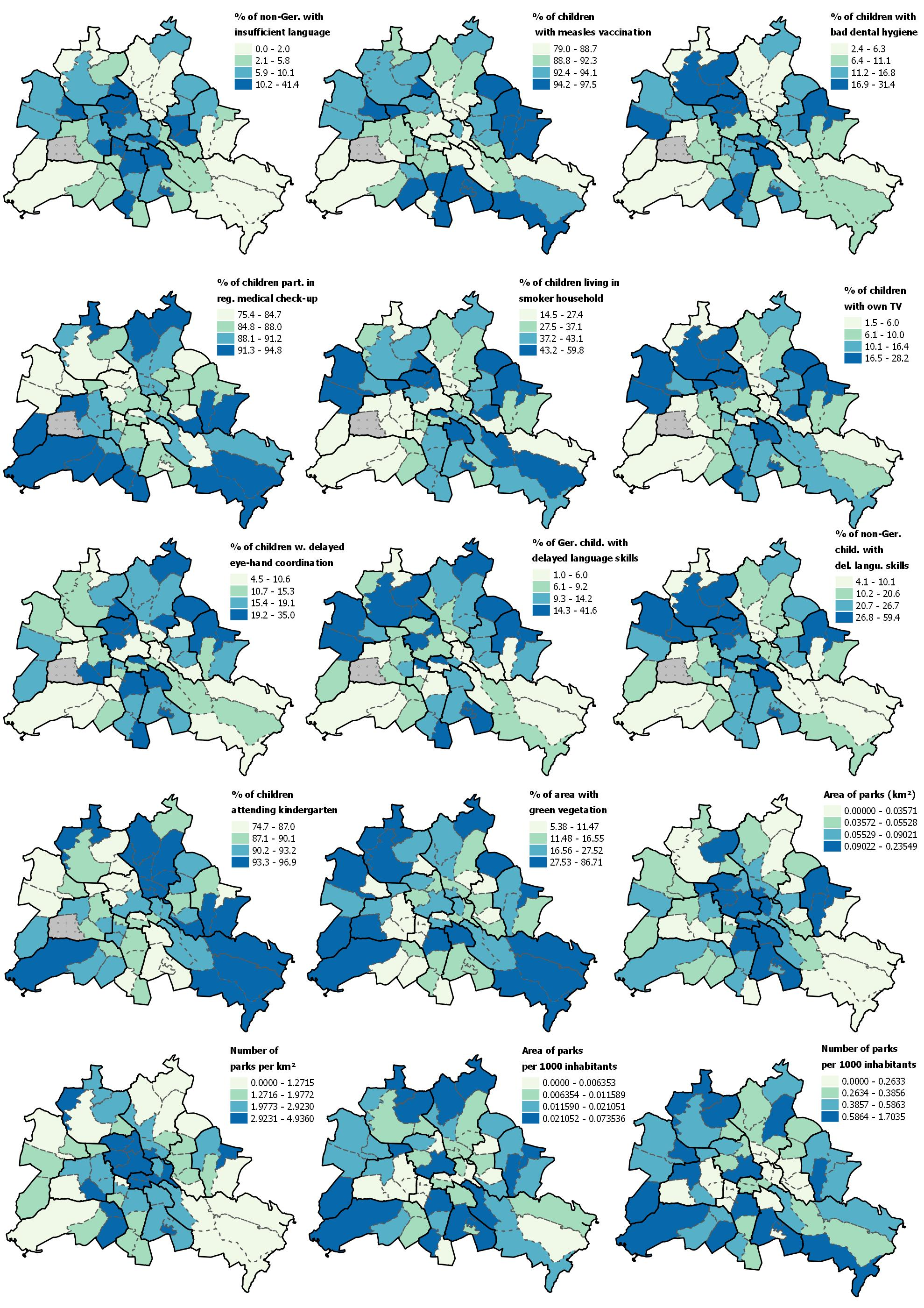

Supplement: Supplementary file 1 — 10.1186/s12942-016-0041-0 Intra-urban patterns of studied influencing factors on overweight and obesity. [file 12942_2016_41_MOESM1_ESM.png]
